# Strategies for SERS Detection of Organochlorine Pesticides

**DOI:** 10.3390/nano11020304

**Published:** 2021-01-25

**Authors:** Rebeca Moldovan, Bogdan-Cezar Iacob, Cosmin Farcău, Ede Bodoki, Radu Oprean

**Affiliations:** 1 Analytical Chemistry Department, Faculty of Pharmacy, Iuliu Hațieganu University of Medicine and Pharmacy, 400349 Cluj-Napoca, Romania; Rebeca.Magda@umfcluj.ro (R.M.); iacob.cezar@umfcluj.ro (B.-C.I.); roprean@umfcluj.ro (R.O.); 2 National Institute for Research and Development of Isotopic and Molecular Technologies, 67–103 Donat, 400293 Cluj-Napoca, Romania; cfarcau@itim-cj.ro

**Keywords:** organochlorine pesticides, POPs, SERS, sensitivity, selectivity, preconcentration, multiplex analysis

## Abstract

Organochlorine pesticides (OCPs) embody highly lipophilic hazardous chemicals that are being phased out globally. Due to their persistent nature, they are still contaminating the environment, being classified as persistent organic pollutants (POPs). They bioaccumulate through bioconcentration and biomagnification, leading to elevated concentrations at higher trophic levels. Studies show that human long-term exposure to OCPs is correlated with a large panel of common chronic diseases. Due to toxicity concerns, most OCPs are listed as persistent organic pollutants (POPs). Conventionally, separation techniques such as gas chromatography are used to analyze OCPs (e.g., gas chromatography coupled with mass spectrometry (GC/MS)) or electron capture detection (GC/ECD). These are accurate, but expensive and time-consuming methods, which can only be performed in centralized lab environments after extensive pretreatment of the collected samples. Thus, researchers are continuously fueling the need to pursue new faster and less expensive alternatives for their detection and quantification that can be used in the field, possibly in miniaturized lab-on-a-chip systems. In this context, surface enhanced Raman spectroscopy (SERS) represents an exceptional analytical tool for the trace detection of pollutants, offering molecular fingerprint-type data and high sensitivity. For maximum signal amplification, two conditions are imposed: an efficient substrate and a high affinity toward the analyte. Unfortunately, due to the highly hydrophobic nature of these pollutants (OCPs,) they usually have a low affinity toward SERS substrates, increasing the challenge in their SERS detection. In order to overcome this limitation and take advantage of on-site Raman analysis of pollutants, researchers are devising ingenious strategies that are synthetically discussed in this review paper. Aiming to maximize the weak Raman signal of organochlorine pesticides, current practices of increasing the substrate’s performance, along with efforts in improving the selectivity by SERS substrate functionalization meant to adsorb the OCPs in close proximity (via covalent, electrostatic or hydrophobic bonds), are both discussed. Moreover, the prospects of multiplex analysis are also approached. Finally, other perspectives for capturing such hydrophobic molecules (MIPs—molecularly imprinted polymers, immunoassays) and SERS coupled techniques (microfluidics—SERS, electrochemistry—SERS) to overcome some of the restraints are presented.

## 1. Introduction

**Figure 1:**
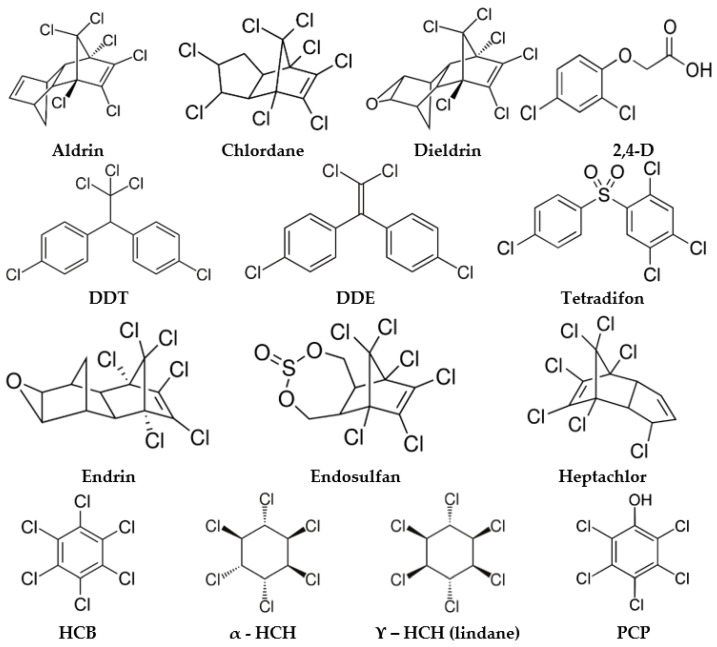
Structures of the listed organochlorine pesticides. DDE—dichloro-diphenyl-dichloro-ethylene; 2,4-D—2,4-dichloro-phenoxyacetic acid.

Organochlorine pesticides (OCPs) are synthetic organic molecules that have multiple chlorine atoms in their structure. They were widely used from the 1940s [1] to the 1960s in the United States and Europe mainly to control insect pests by affecting their nervous system [2]. Most OCPs are lipophilic and hydrophobic (Figure 1), and as such, they are very resistant to environmental degradation [3], persisting in the environment for years or decades after usage. This leads to the contamination of groundwater, surface water, food products, air, and soil. An extensive literature review [4] of data from North America, Europe, and Asia on emerging pollutants in water sources identified organochlorine pesticides as one of the most common water pollutants. Due to the great health risks posed by OCPs at environmental concentrations, most of them are included in the group of contaminants known as persistent organic pollutants (POPs).

As the negative impact of pesticide pollution transcends national boundaries, POPs have been regulated by the International Agreement (United Nations Environment Program, UNEP) between 154 signatories of the Stockholm Convention on Persistent Organic Pollutants since 2001. Nine OCPs, namely aldrin, dieldrin, endrin, dichloro-diphenyl-trichloroethane (DDT), chlordane, hexachlorobenzene (HCB), mirex, toxaphene, and heptachlor, were initially listed amongst the 12 initial POPs [5] called the dirty dozen. Since then, chlordecone and hexachlorocyclohexanes (HCH) including lindane (γ-HCH), endosulfan (α and β isomers), dicofol, pentachlorophenol (PCP) and pentachlorobenzene have also been listed [6]. These chemicals are dangerously toxic pollutants, capable of long-range transport, bio-accumulation in human and animal tissues, and bio-magnification in food chains [7].

Many studies keep finding associations between human long-term exposure to OCPs and a large panel of common chronic diseases including cancer (breast, prostate, testicular, kidney, ovarian and uterine cancers), neurodegenerative diseases (Parkinson, Alzheimer, Amyotrophic Lateral Sclerosis), chronic respiratory diseases (asthma and Chronic Obstructive Pulmonary Disorder), diabetes, immune dysfunction, cardiovascular diseases, endocrine disruption, and even harmful reproduction effects [8,9,10]. There are some recent reviews that cover this topic [11,12].

Even if great efforts are made to phase POPs out globally, with obvious progress, they are still found in the environment in regions such as the Arctic [13], thousands of kilometers from any major POP source. Moreover, as novel data are gathered, the list of EPs or environmentally persistent hazardous chemicals is continuously amended. Most recently, in June 2017, the Stockholm Convention established global bans on 16 new POPs [14] including organochlorine pesticides. This translates into financial (expensive equipment) and technical challenges (time-consuming purification processes) regarding the scientific analysis of the samples given that they are conventionally analyzed by chromatographic techniques such as gas chromatography coupled with mass spectrometry (GC/MS) or electron capture detection (GC/ECD) and HPLC-MS [15,16,17]. Even if the detection and quantification of analytes at trace levels is accurate down to ng or pg/L, these techniques can only be used in well-equipped centralized laboratories, once the sample is collected and extensively pre-processed. In this context, accessible new strategies for inexpensive, fast, highly sensitive, and on-site detection of OCPs such as surface enhanced Raman spectroscopy (SERS) are of very high demand. The current review focused on strategies adopted to maximize the SERS signal of OCPs. This can be achieved by increasing the substrate’s performance by increasing the affinity of the analyte for the substrate or by preconcentration. Strategies like concentrating nanoparticles through mechanical traps [18], inducing hot-spots by aggregation or assembly of nanostructures [19] in different-sized oligomers, films, or film patches [20] are all improving the sensitivity of the substrate. Such approaches may even enable the detection of molecules with low affinities toward the metal substrate.

While maintaining their sensitivity, the selectivity of the SERS substrates may be further tuned by functionalizing them with linkers such as diamine [21,22] dithiols [23], bipyridinium dications [24], carbon, and metal-organic-frameworks [25], to name a few. The interaction of these “receptor” molecules with OCPs via covalent, electrostatic, or hydrophobic bonds will be discussed. Moreover, the potential advantages of multiplex analysis and future prospects are also presented.

## 2. Brief Introduction to Surface Enhanced Raman Spectroscopy (SERS)

The inelastic scattering of photons by molecules, discovered in 1928 as “a new type of secondary radiation” [26], is known as Raman scattering. This effect is very weak, specifically, there is an inelastic scattering for every ten million elastically scattered photons. As Raman spectroscopy provides the great advantage of a molecular fingerprint (in particular, the molecule’s vibrational structure), it was fortunate that in 1974, researchers developed discovered methods to amplify the weak Raman signal [27]. This is when SERS emerged. The SERS effect denotes a strong increase (several orders of magnitude) in the Raman signal of a molecule, induced with the help of a special substrate, typically represented by metallic nanostructures.

The enhancement of the Raman signal by the metal substrate is explained mainly by an electromagnetic mechanism cumulated or not with a chemical one [28,29]. At a certain resonant frequency, the interaction of electromagnetic radiation with metal nanostructures leads to collective oscillations of the conduction electrons (i.e., the excitation of surface plasmons (plasmon resonance)). One of the main consequences of this resonant plasmon excitation is the strong enhancement of the electromagnetic near fields at the metal surface. It is this resonantly enhanced near field that is the main contributor to the Raman signal enhancement in SERS. Since near fields decay exponentially away from the surface, it has been established that it is critical for the molecule to be in close proximity of the metallic surface (furthermost 10 nm) [28] to increase the Raman signal. The enhancement is maximized for molecules in direct contact with the surface, and it decreases with the increase in distance between the substrate and the analyte. By matching the surface plasmon resonances (localized or propagative) to the excitation laser, which can be done by material, size, and shape adjustments, the SERS efficiency of a substrate can be maximized at the desired wavelength. To extend SERS effects into the deep-UV and benefit at the same time from the resonance Raman effect (i.e., matching molecular electronic transitions), aluminum SERS substrates have been developed [30].

The SERS enhancement based on the electromagnetic mechanism applies to all molecules and leads to enhancement factors (EF) as high as 10^6^–10^8^. The chemical mechanism, on the other hand, is based on charge transfer interactions that take place between molecules and the metal surface. This requires the molecule to be chemically adsorbed on the surface of the metal substrate, making this mechanism analyte-dependent and site-specific. Molecules can be adsorbed on the surface either through physisorption (van der Waals forces) or chemisorption (chemical bonds such as covalent or electrostatic interactions).

Since it provides a broad range of advantages, SERS represents a valuable analytical tool for the detection of pesticides. Among these, the high sensitivity and the generated vibrational fingerprint of molecules are highly valued since OCPs are found in trace amounts in rather complex matrices of the environmental samples and they can have multiple stereoisomeric configurations. Moreover, the analyses are fast (given that there is no need for thorough purification processes), the equipment is not very costly, and it is easy to use. Additionally, there are portable Raman spectrometers for on-site analysis.

As every analytical technique, it has also its drawbacks, certain concerns in the SERS analysis (of pesticides) involving selectivity, sensibility, reproducibility, portability, quantification, and nonspecific binding. These challenges have been the subject of multiple comprehensive reviews [31,32,33], consequently they will not be reconsidered in detail. Bernat et al. [34] presented limitations like selectivity, reproducibility, and nonspecific binding, along with some possible solutions. Reviews regarding different spectroscopic techniques (SERS, SPR, and fluorescence) for detecting POPs [35] or pesticide residues in foods [36,37] can also be found in the literature. Some recent book chapters have addressed more general matters like usage, sensing (exploiting biofunctionalized nanomaterials), and removal of different classes of pesticides (organochlorine, organophosphate, carbamate, pyrethroids and others) [38] or development of optical (including SERS) and electrochemical sensors for pesticide detection [39].

OCPs are hardly mentioned in these works as their SERS spectrum is more troublesome to be obtained because of their highly hydrophobic structures (Figure 1) and low affinity toward the SERS substrates. Additionally, the important progress made in the last few years regarding their analysis has built the momentum of a more comprehensive discussion and review on the SERS detection strategies of OCPs, as summarized in Table 1.

## 3. Increasing the Electromagnetic SERS Enhancement

Typically, SERS substrates are represented by noble metal nanostructures, like Au, Ag, or Cu. They come in a large variety of structures included in multiple categories such as liquid-phase metallic colloids and their aggregates/assemblies, and solid substrates consisting of nanoparticle films/sheets, or patterned surfaces fabricated by electrochemical techniques or different variants of nano-lithography. More details on SERS substrates may be consulted from several reviews on this topic [59,60,61,62].

Gold and silver colloidal nanoparticles are classic examples of SERS-active substrates. They are stable for a long period of time, they are easy to synthesize, and exhibit good to excellent SERS performance. In colloidal solution, the magnitude of the SERS enhancement is strongly dependent on the shape, size, and surface morphology of the nanostructures. These parameters allow for tuning of the plasmon resonances, and thus enable a rational selection of wavelengths at which electromagnetic fields and SERS will be maximized. Then, nanostructures with sharp features like stars, cubes, prisms, or nanoplates generate high electromagnetic fields at their tips or along edges, known as the “lighting rod effect”. Consequently, they exhibit improved SERS performance compared to other structures with smooth surfaces like nanorods, nanobars, or nanowires and are more suitable for ultrasensitive detection. Alternatively, ultra-small, few-nm wide gaps between metallic parts are desired for high electric field enhancements including the so-called “plasmonic antenna effect” [63].

However, investigating organochlorine pesticides in colloidal solution is problematic in complex matrices such as environmental samples, since the stability of the constituting nanostructures is based on electrostatic repulsions and can be disrupted by a large variety of molecules/bacteria/residues and by pH variations. This leads to random aggregation and poor reproducibility of the signal. Moreover, molecules that are unable to interact with the plasmonic surface are hardly detected. For example, multiple authors have reported that without functionalization, they could not record a SERS spectrum of OCP molecules [21,22,24]. As an exception, the direct detection of 2,4-D, an organochlorine herbicide, could be achieved in colloidal samples of gold nanorods and nanocubes [40]. This was possible because it has a more water friendly structure due to its carboxylic moiety.

A quest for non-noble metal substrates for OCP detection has also recently started [49], even if still in its infancy. Commonly, they have been investigated as SERS substrates as they would expand the range of materials and possibilities for surface functionalization used for this technique [64]. Non-noble metal SERS substrates are typically limited by inferior sensitivity and sometimes instability (oxidized under laser irradiation or in contact with corrosive materials). Colloidal MoO_2_ represents a relatively new SERS substrate material that falls under the category of semiconductors with the surface plasmon resonance effect attributed to the presence of oxygen vacancies. Zhang et al. developed MoO_2_nanodumbbells via a simple hydrothermal method, with surprising high stability and oxidation resistance as analytical platforms for SERS detection of PCP and other high-risk chemicals like bisphenol A and dichlorophenol. Moreover, this material presents a sensitivity comparable to that of Au or Ag substrates [49].

**Figure 2:**
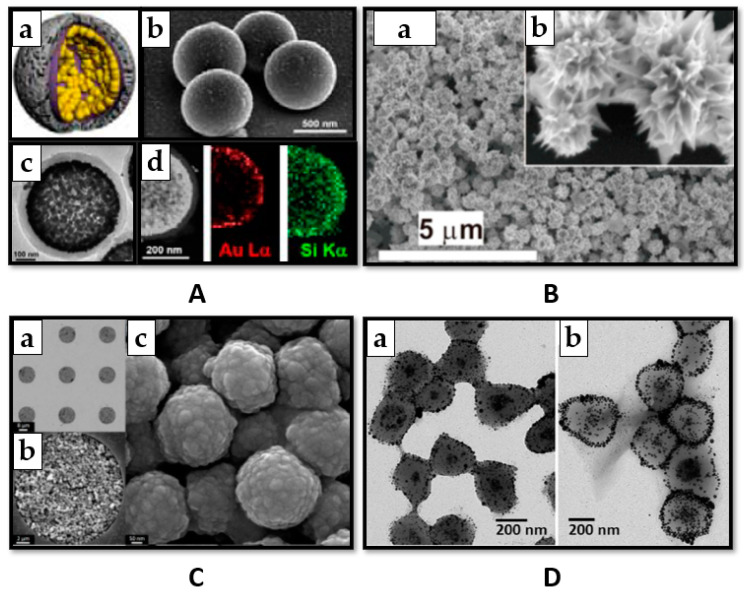
Strategies for increasing the electromagnetic surface enhanced Raman spectroscopy (SERS) enhancement. (**A**) (**a**) Schematic illustration of the capsule; (**b**,**c**) Representative TEM micrographs of the capsules; (**d**) STEM/XED analyses of the capsule composition. Reproduced with permission of [42]. Copyright John Wiley and Sons, 2019. (**B**) (**a**) FESEM image of the chestnut-like Au nanocrystal film, (**b**) local magnified image. Reproduced with permission of [19]. (**C**) (**a**–**c**) SEM images of F-NPs substrate with 100 nm of Ag coating film. Reproduced with permission of [47]. Copyright IOP publishing, 2020. (**D**) Representative TEM images of (**a**) the Fe_3_O_4_@pNIPAM nanohybrid materials containing Ag seeds; (**b**) the finalFe_3_O_4_xAgNPs@pNIPAMcomposite microgels. Reproduced with permission of [50]. Copyright American Chemical Society, 2011.

Meanwhile, there is an ongoing pursuit for new colloidal SERS substrates adapted for detecting and quantifying target analytes. With the scientists’ ingenious design of nanostructures, the possibilities are limitless. For instance, Mariño-Lopez et al. (2018) developed an original SERS substrate for DDT detection and ultrasensitive quantification in water samples. They synthesized microporous silica capsules inner-coated with a layer of gold nanoparticles (Figure 2A). Large electromagnetic fields have emerged from interparticle hot spots and thus DDT could be directly detected from minute amounts added to the colloidal dispersion. This unique microporous structure exhibited exceptional colloidal stability compared to colloidal gold nanoparticles, which aggregated and precipitated within 20 min of exposure to the analyte. Moreover, in natural water samples, the capsules possessed a certain degree of selectivity, with the microporous shell acting like a sieve for large molecules and microorganisms [42].

Another strategy for increasing the substrate’s performance is loading gels with nanostructures and mechanically trapping analytes upon collapse. This occurs upon drying or exposure to high temperature, offering the potential of generating dynamic interparticle nanogaps, or hot-spots. Nevertheless, the surrounding polymer shell may hinder the efficient formation of hot-spots, restricting the achievement of very low detection limits. The gel can be rehydrated to release the molecules that were trapped, thus having recyclable properties. Using this approach, Aldeanueva-Potel et al. reported a composite agarose gel highly loaded with silver nanoparticles for the SERS detection of DDT, which could be trapped in the network and detected from aqueous solutions with considerable sensitivity [18]. Nanostructures embedded in gels can be even further concentrated using magnetic functionality. Contreras-Caceres et al. reported, for the first time, the SERS detection of PCP in trace amounts by encapsuling both silver and magnetic nanoparticles in a poly(N-isopropylacrylamide) microgel (pNIPAM) (Figure 2D). This translated in improved detection limit for SERS measurements and considerable economy in substrate manufacturing [50].

A very efficient method for enhancing the SERS signal is represented by aggregation. Aggregating individual nanoparticles leads to the formation of interparticle hot-spots and thus, an even stronger electromagnetic field. This phenomenon occurs when strong electrolytes (i.e., NaCl) are added to a colloidal solution and disrupt the colloidal stability or when the analyte itself displaces the stabilizing agents from the metallic surface. Spontaneous aggregation of citrate reduced gold nanoparticles after exposure to endosulfan has been reported. Even if it is known that organochlorine pesticides have low affinity for metallic substrates (especially charged ones), the authors reported the formation of aggregated micrometric structures with irregular shapes after incubation with different concentrations of endosulfan in 2-propanol for 9 h [46].

Aggregating or assembling nanostructures in films leads to more stable substrates and increased SERS efficiency. Drying is the most facile method for achieving this. If the sample is not analyzed in a SERS cuvette, and instead, small volumes are drop-casted on a microscope slide, evaporation can occur during laser excitation. This leads to poor reproducibility of the experiments and drying is usually applied before analysis as an answer to this problem. This technique was reported by Zhou et al. for the analysis of lindane. First, chestnut-like Au nanocrystals were synthesized, presenting significant electromagnetic field enhancement at the tips and gaps between the nanoneedles (120–150/nanocrystal). Next, the colloidal nanostructures were dropped on silicon and dried, creating a ~500 nm thick film (Figure 2B) with even better SERS performances. Tested on lindane, a limit of detection (LOD) of 10 ppb could be achieved [19].

Given a good occasion to observe the differences in the enhancement capabilities of nanostructures with different morphologies, the same authors used trisoctahedral and calyptriform Au nanocrystals to build films in the same manner, reaching a LOD for lindane of only 30 ppb [48].

Magnetic force could also assist in the self-assembly of nanostructures in even closer packed films. In this regard, Gong et al. developed a 3D substrate of silver coated ferro-nanoparticles (F-NPs) in previously manufactured microarrays (Figure 2C). The tight bounded structures, along with the vertical stacking and the nano-protrusions on the surface, generated strong hot-spots suitable for sensitive SERS analysis of pesticide residues, such as HCB from natural soils. The magnetic force-assisted 3D self-assembly of nanoparticles generated SERS substrates with superior performance to the ones prepared without magnetic force and 35 times better than single-layered F-NPs [47].

The applicability of the SERS substrates can be further improved by solving the issue of inter-batch reproducibility of the substrate. Employing nanoimprint lithography, electrochemical or laser assisted methods could help manufacture reproducible patterns. Kim et al. used nanoimprint lithography to fabricate high-density and uniform arrays of gold nanofingers [65] that could trap pesticides between their tips after exposure to the analyte. Applying this novel technique on chlorpyrifos, an organophosphate pesticide with chlorine atoms, the interactions with the gold fingertips was put on the account of either the phosphate or chlorine groups. Even if this platform could simplify and accelerate the trace detection of this pesticide, applied on organochlorine derivatives might lead to less satisfactory results.

Electrochemical techniques for SERS substrate manufacturing have been explored for features like high stability, large surface areas, and low manufacturing costs. Applying repetitive oxidation–reduction cycles on metallic electrodes leads to roughening of the surface (thereby this process is named electrochemical roughening). By varying the electrochemical parameters or the type of electrolyte, it is possible to optimize the SERS performance of these substrates. Spencer et al. described a silver oxide SERS sensor via electrochemical roughening [51], applied for the rapid field analysis of a large variety of pesticides including organochlorine compounds in both the liquid (artificial urine samples) and vapor phase. Chi et al. synthesized a nanoporous silver sheet by reduction-induced decomposition [45], developing multiple layers with a delicate, coral-rock-like morphology. As such, the range of analytes is no longer limited to molecules with a high affinity toward the metal surface. For example, ubiquitous organic pollutants such as lindane and DDT could be conveniently quantified regardless of their weak affinity toward the substrate. Additionally, the substrate could be regenerated after ultrasonic cleaning in water and reused for at least 20 times. Zhu et al. developed a binary-template-assisted electrodeposition method to fabricate highly ordered arrays of vertically aligned Ag nanorod bundles. The resulting substrate showed good reproducibility, along with a SERS EF as high as 10^8^ due to the narrow gaps of 2 nm formed between nanorods and has been applied for the detection of 2,4-D and some other pollutants.

Since highly ordered arrays of nanostructures can be manufactured by laser-assisted methods, this technique is convenient for fabricating highly reproducible SERS films with the use of low-cost materials. Moreover, their optical properties, and consequently their SERS performances can be easily adjusted by modifying the processing parameters. Nedyalkov et al. investigated different laser-assisted methods (conventional pulsed laser deposition of metal films further modified by nanosecond pulses and femtosecond pulsed laser deposition in air) for the synthesis of two- and three-dimensional metal nanostructure films [43]. They successfully tested the substrates for active SERS detection of DDT, along with other pollutants (nitrates) or Raman reporters (methylene blue).

However, the performance of SERS substrates, as characterized by electromagnetic enhancement factors, can only be increased to a certain degree. For hydrophobic molecules, like organochlorine pesticides, the lowest LOD was achieved by integrating a magnetic functionality in the SERS platform, being situated in the nanomolar range for PCP [50]. For comparison, the authors registered with the same substrate, a picomolar LOD for molecules with high affinity toward the metal substrate such as 1-naphthalenethiol.

## 4. Increasing the Affinity toward the Analyte

Modulating only the substrate’s performance has proven to be insufficient for ultra-trace detection of lipophilic molecules. Even if hot-spots are generated, analytes must be able to reach these sites, on or very close (<10 nm) to the metal surface.

Chemical functionalization of the metal surface is able to drive analytes closer to the substrate’s surface in regions where the intensity of the electromagnetic field is strong, thus leading to maximum SERS enhancement. Since the sensitivity of detection is directly affected by the adsorption efficiency of the targeted molecule, efforts are directed toward rational surface functionalization. In order to increase the interactions between pesticides and nanostructures, intermediate linker molecules are used. These possess functional groups in their structures such as thiol, amine, hydroxyl, and carboxyl, which make covalent, hydrophobic, or electrostatic interaction with the analyte possible. The ideally-functionalized substrate would facilitate the specific binding of one class or even a single analyte of interest. In what concerns OCPs, the analysis of the whole class in a single run would be useful since multiple compounds are often present in the same sample.

**Figure 3:**
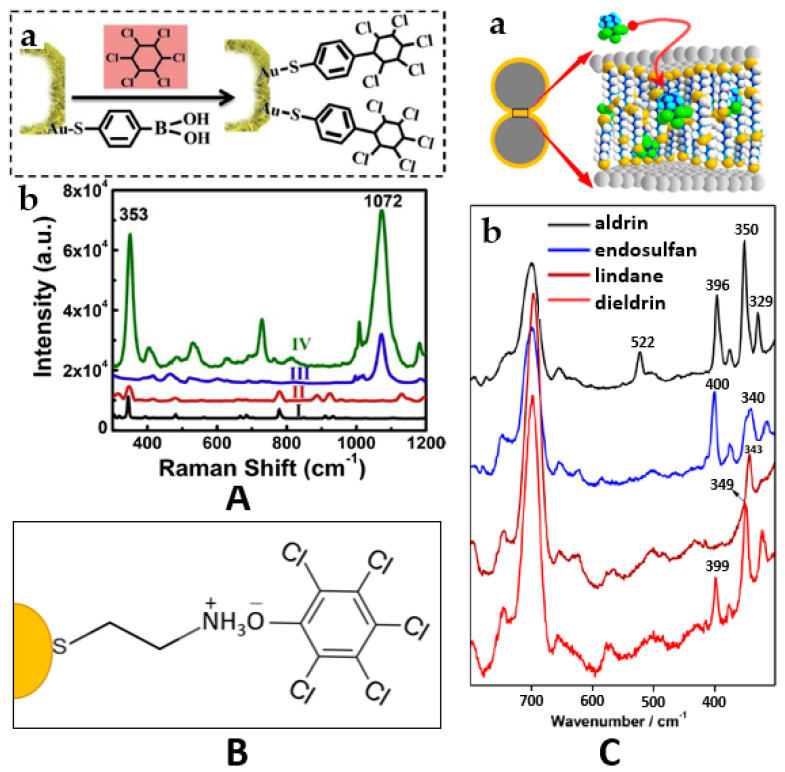
Strategies for increasing the affinity toward the analyte. (**A**) (**a**) The Suzuki cross coupling reaction occurred between HCH and 4-MPBA via covalent linkage (C–C) on the modified substrate. (**b**) The Raman spectra of solid HCH (curve I), the SERS spectra of γ-HCH (10^−6^ M) obtained on the naked ANHC substrate (curve II), 4-MPBA modified ANHC substrate (curve III), 4-MPBA modified ANHC substrate immersed into 10^−6^ M HCH solution (curve IV). Reproduced with permission of [53]. Copyright Elsevier, 2019. (**B**) Schematic representation of the mechanism of sensing PCP using cysteamine modified substrates. (**C**) (**a**) Scheme displaying the pesticide hosting (in this case, for aldrin) in the dithiol layer organized in interparticle gaps; (**b**) SERS spectra of the analyzed pesticides (10^−5^ M) on DT8-functionalized AgNPs, showing the C–Cl stretching bands of the fingerprint region (300–400 cm^−1^) and the C–S stretching bands of DT8 in the deduced multilayer highly ordered conformation employed as the reference band for quantitative analysis. Reproduced with permission of [23]. Copyright American Chemical Society, 2015.

As a first example, 4-mercaptophenylboronic acid (4-MPBA) was used by Zhou et al. as a bifunctional covalent linker between the synthesized Au nanosheet hollow sub-microcubes (ANHCs) and the HCH, as the analyte. The interaction with the substrate was on the account of Au–S covalent bonding, while the interaction with the analyte occurred via Suzuki cross coupling between the linker’s boronic acid group and the halogenated hydrocarbon in optimum conditions of pH 7 and a temperature of 80 °C. The magnification in the SERS signal was attributed first to the gaps and edges in the cross-arranged nanosheets, which generated a strong electromagnetic field. Being chemically bound in close proximity to the metal nanostructure, HCH molecules could profit from this enhancement (Figure 3A). Furthermore, differentiation of mixed HCH isomers in water samples could also be achieved [53].

Another common mechanism for analyte attachment is electrostatic interaction. It is worth mentioning that only molecules with opposite charge can be adsorbed and most OCPs have no ionic/ionizable functional groups. A molecule frequently used for this purpose is cysteamine. This molecule has some intrinsic advantages such as the bifunctional bonding forming a covalent linkage with the metallic substrate via the S atom and an electrostatic interaction with the analyte via the amine group. Moreover, it forms a self-assembled monolayer (SAM) on the surface of nanostructures, leading to uniform adsorption of analytes. Another advantage of this linker molecule is the short carbon chain (two atoms), which allows the adsorption of analytes in close proximity to the metal surface, leading to stronger SERS enhancement. Several groups of researchers have reported this strategy for the detection of PCP because of its phenolic functional group available for binding with this linker (Figure 3B). Jiang et al. used silver nanoparticle aggregates on a copper foil as SERS active substrates [54], while Ma et al. used gold nanoparticles covalently immobilized on a glass substrate [55]. Bian et al. voltammetrically-synthesized a cysteamine-modified porous silver fiber as a SERS substrate, which could also be used for the extraction of the organochlorinated pesticide via solid phase microextraction [56]. Cysteamine could electrostatically interact with PCP and preconcentrate it on the stationary phase, with the additional possibility to quantify the adsorbed analyte. Since quantification is usually a challenge in SERS spectroscopy [66], an internal standard adsorbed on the nanostructure’s surface can readily solve this issue [33]. In all occasions, upon the functionalization with cysteamine by SAM formation, the characteristic Raman bands of the linker were also used as an internal reference. Since the Raman signal of the standard is exploited to normalize the Raman spectrum of analytes, experimental variability induced by sample positioning, focusing, or laser power fluctuations may be marginalized [67].

If some molecules, like PCP, may engage in electrostatic interactions through the phenolic function, most OCPs do not present this advantageous feature (Figure 1). Consequently, these molecules can only be adsorbed via hydrophobic interactions. In this context, diamine or dithiols could be used as custom SAMs that would adsorb molecules and additionally, protect the substrate.

Aliphatic diamines are known to generate SAMs on the surface of metal nanostructures. If the alkyl chain of the diamine is too short (2 C atoms), there is not enough space to fit the hydrophobic molecules (similar to previously discussed cysteamine) and when the alkyl chain is too long (12 C atoms), the SAM is stabilized by strong intermolecular interactions, and thus the analyte cannot be included between diamines. Guerrini et al. demonstrated that diamines with an intermediate chain length (8 C atoms) interacted best with organochlorinated lipophilic molecules [21]. They used 1,8-diaminooctan as a bifunctional molecular linker to mediate the interaction between silver nanoparticles and produce dimers with tuned interparticle distance. The adsorption of diamines on the metal surface via ionic interactions was mediated by chloride ions. The performances of the SERS substrate were demonstrated on aldrin, which hydrophobically adsorbed within the SAM, and the signal enhancement was attributed to the large interparticle electromagnetic fields. The same group of authors also reported the detection of α-endosulfan and β-endosulfan with the same SERS substrate [22]. For these molecules, apart from the hydrophobic interactions, electrostatic interactions (ion pairing) emerged between the pesticides’ vicinal chlorine atoms (from the unsaturated carbon atoms of the benzodioxathiepinic ring) and the positively charged groups of the diamines. The authors emphasized that this substrate could mimic the biological target of organochlorine pesticides (since they interact with the neuronal cell’s membrane). Even so, as stated at the beginning of this chapter, for maximum SERS enhancement, molecules have to be adsorbed in the hot-spot gaps generated between the nanoparticles. Even though a restrictive film forming process is difficult to control, forming a SAM on the whole particle surface would only lead to random interactions in any area.

Alkyl dithiols have similar properties with the previously discussed diamines, forming SAMs on the surface of nanoparticles. They induce nanoparticle aggregation and provide hydrophobic interparticle junctions where the electromagnetic field is strong and the chlorinated target molecules can be adsorbed via van der Waals and/or hydrophobic forces (Figure 3C). Consequently, functionalizing nanoparticles with alkyl dithiols (like diamines) can help in improving both the substrate’s performance and the affinity of several organochlorine pesticides to the metal surface. Kubackova et al. studied the effect of different experimental conditions on the resulting SERS substrate and concluded that the maximum magnification of the pesticides’ SERS signal could be achieved using silver nanoparticles (rather than gold nanoparticles) functionalized with 1,8-octanedithiol (DT8) (rather than other aliphatic or aromatic dithiols with different chain length) [23]. The alkyl chains of dithiols are claimed to be organized in multilayers under these conditions, providing a maximum number of binding sites at the formed hot-spots localized in the gaps between the bridged nanoparticles. Different detection limits were found for the distinct organochlorine molecules aldrin, dieldrin, lindane, and α-endosulfan due to their different affinities toward the functionalized substrate (Figure 3C).

Aromatic viologen compounds (bipyridinium dications) attract considerable attention due to their three interesting reversible redox states. Exhibiting these properties, they are studied as functional materials for a wide range of applications such as electrochromic devices, molecular machines, and organic batteries [68,69]. One of the less exploited applications is their use as bridge molecules. Viologens can strongly adsorb via the quaternary amines on the metal surface after exposure to halide ions and interact with both the nanostructures and the hydrophobic analytes (polycyclic aromatic hydrocarbons, organochlorine insecticides), bringing them in close proximity for improvement in the SERS signal [70]. In 2008, Guerrini et al. used lucigenin to functionalize silver nanoparticles and reported for the first time, a SERS spectrum of endosulfan [24]. Endosulfan was admixed upon the synthesis of the silver colloid, followed by the addition of lucigenin and the subsequent activation of the mixture with chloride ions. This induces nanoparticle aggregation and thus, an increase in the SERS signal. Moreover, chloride ions promote the adsorption of lucigenin by forming a charge transfer complex and lead to an increase in SERS signal by chemical enhancement contribution [71]. Regardless of its poor affinity toward the plain metallic substrate, the surface adsorption of endosulfan on the nanoparticle’s surface occurs due to a chemical interaction involving both parties. Namely, α-endosulfan converts into β-endosulfan upon adsorption between the lucigenine molecules and forms a charge–transfer complex (chlorine atoms from the unsaturated benzodioxathiepinic ring of endosulfan with the N atom of the lucigenin’s central acridinium ring). At the same time, viologen suffers a rotation of the acridinium planes in order to adjust the pesticide molecule. Authors state that other structurally-related molecules, like aldrin, do not interfere with the above reaction, rendering the reaction highly specific for endosulfan. However, endosulfan could not be detected when related molecules were used for functionalization (paraquat or diquat). Nevertheless, Zhang et al. functionalized flower-like silver nanoparticles with either diquat or lucigenin [52]. Chloride ions were used for the same reasons as above and nanoflowers were further aggregated by droplet evaporation. The substrates were tested on different organochlorine molecules like α-endosulfan, β-endosulfan, op’-DDT, HCH, and aldrin. Even if the resulting SERS intensities were not so strong, the use of viologen remains a valid strategy in the SERS detection of organochlorine pesticides. The authors emphasize that using diquat or lucigenin as bridge molecules can bring a certain degree of selectivity to the analysis. Diquat was suitable for the majority of the analytes (including endosulfan), while lucigenin only for several representatives (op’-DDT and aldrin). This was attributed to the diversity in the analytes’ binding energy.

Another interesting and newly-reported strategy for the detection of OCPs (and several other POPs) is carbon-assisted adsorption on the surface of noble metals, reported by An et al. [57]. The SERS active substrate was synthesized by depositing a layer of silver nanoparticles on carbon-coated Fe_3_O_4_ microspheres. They exploited the hydrophobic nature of carbon within the SERS substrate composite, having a strong affinity toward the aromatic nucleus of the analytes and facilitating their adsorption near the silver surface. More than being exceptionally SERS active, the magnetite-carbon core shell microspheres coated with silver nanoparticles have superparamagnetic properties that allow their close packing into a small volume. Thus, multiple hot-spots are generated and excellent SERS enhancement with LOD down to the picomolar range is achieved. This type of substrate is also advantageous because due to its magnetic functionality, it can be easily cleaned from residual reagents of the fabrication processes, resulting in a more efficient adsorption of analytes.

Another efficient approach is the use of metallic organic frameworks (MOFs). They are hybrid crystalline materials with high porosity and large internal surface area. In addition, they present other advantages like stability or high selectivity [72]. They are represented by versatile structures with adjustable properties used for many applications. Combined with nanostructures, they become suitable for SERS applications. Interestingly, the SERS effect of non-modified MOFs (no integrated metallic colloids) has also been reported [73]. Zhou et al. reported the use of such substrates for the detection of α-HCH and γ-HCH [25]. They synthesized urchin-like Au–Ag nanocrystals with high SERS performance due to the sharp and dense edges. These nanostructures were then wrapped in a porous zeolite imidazole framework, which allowed HCH molecules to be caught close to the substrate. This core shell configuration has a certain degree of selectivity since the pore sizes would only allow the adsorption of molecules smaller than 11.6 Å.

A relatively long period of incubation with the analyte is needed in order to reach an equilibrium state. Adsorption kinetics vary between the target molecules, dependent on their molecular structures and the substrate’s nature. Some studies, evaluating the influence of the analyte’s incubation time with the substrate revealed a negative correlation with its concentration. At higher PCP concentrations (10 µM), the adsorption reached saturation after 6 min, whereas at lower concentrations (1 and 0.5 µM), the saturation time was much longer (68 and 113 min) [54]. Other studies showed that the adsorption equilibrium on another substrate for the same target molecule of different concentrations was achieved in 175 min [56].

As can be seen, apart from optimizing the nature, shape, and morphology of the SERS substrate for higher enhancement factors and improved reproducibility, noteworthy additional benefits are promoted by rational substrate functionalization in terms of OCP detection sensitivity and selectivity.

## 5. Sample Processing and Preconcentration

An ideal SERS analysis of pesticide residues would require minimal sample preparation and a short analysis time, which could be performed on-site, in a decentralized manner. While p,p’-DDT and some other pesticides can be detected rapidly from the surface of an apple using a flexible SERS substrate and a simple wiping technique [44], it is hard to detect organochlorine pesticides in complex environmental matrices. Real environmental samples are more complex and encompass a broad range of chemicals and microorganisms, part of which would compete for adsorption on the SERS substrate. This becomes a real problem in trace analysis. This is why most SERS experiments stop at spiked samples and do not demonstrate real applications in complex matrices. For surface waters, a physical and/or chemical filtration could be applied in order to simplify the sample matrix. Designs of SERS substrates like core-shell microporous capsules [42] or MOFs [25] have been reported to work as filters for larger molecules in the detection of organochlorine pesticides from water samples. Samples like fruits [74], vegetables, or soil may not have a uniform distribution of the pesticides and thus, sample processing is needed for a valid detection [75]. Moreover, some pesticides may form complexes with proteins or other matrix constituents, which would also imply sample pretreatment. Even if extraction is usually a time-consuming step, it is most often needed for real environmental samples as it brings additional benefits such as analyte preconcentration, cleaner background, and easier identification of analytes. Moreover, much simpler sample preparation procedures are required for SERS analysis in comparison with those for liquid chromatography or GC-MS, because only the laser comes into contact with the sample. Elaborate extraction procedures commonly used in chromatographic techniques are not needed in SERS analysis since the SERS substrate itself may efficiently fulfill class selective- or even specific analyte preconcentration. For example, Bian et al. designed a cysteamine-modified porous silver fiber substrate that could be used for both solid phase microextraction of PCP from water samples and its SERS detection [56].

A conventional liquid–liquid extraction of organochlorine pesticides in organic media, followed by solvent evaporation has also been reported. Such an approach would separate the analytes of interest from all water soluble interferents that may have a higher affinity for the unmodified SERS substrates. Concentrating the analyte leads to a higher probability of adsorption and thus, a higher SERS signal. Qu and He [58] described a simple “rolling” method for detecting chlordane in livestock oil with the help of citrate-coated gold nanoparticles, which usually demonstrate weak interaction with hydrophobic organochlorine pesticides. This method relies on mixing a methanolic solution of chlordane (which resulted after its extraction from oil samples) with colloidal gold nanoparticles with the help of a pipette on a piece of parafilm. Once half the volume of solvents is evaporated, another batch of chlordane in methanol is added. This is repeated multiple times in order to preconcentrate the analyte and achieve the adsorption of chlordane on the negatively charged gold nanoparticles. Afterward, full solvent evaporation is triggered in order to further improve the interactions between analytes and nanoparticles. As a result, the SERS signal of molecules with low affinity to the surface of metal nanoparticles is efficiently enhanced. Combined with a mathematical prediction model, the “rolling” method is suitable for quantitative analysis of hard-to-detect analytes from complex food samples. The authors stress that this method is not suitable for detecting molecules with strong affinity for the surface because the rolling step could promote the aggregation of nanoparticles and thus, the quenching of the signal.

## 6. Multiplex Analysis

Multiplex analysis refers to the detection of multiple analytical targets from the same sample at the same time. This strategy would rapidly reveal complex information about the chemical composition of the environment. Detecting different molecules (from the same or distinct structural classes) at the same time is common for chromatographic techniques after extraction and purification. For example, the detection of 14 organochlorine pesticides in soil samples [76] or 23 POPs in human plasma, among which five organochlorine pesticides [77] were reported.

SERS shows great potential for multiplex analysis [78], but this is hardly reported for organochlorine pesticides and even less common for stereoisomers. Ma et al. [79] self-assembled unmodified gold nanoparticles (AuNPs) at a cyclohexane/water interface after exposure to the analytes, stating that this method could be used for trapping molecules (regardless of their hydrophilic, hydrophobic, or amphiphilic structures) from different liquid phases, resulting in multiplex detection. While this is possible in theory, competition for adsorption on the metallic surface could take place and the affinity for the SERS substrate of different molecules in the same mixture might be modulated by their concentration, which would make SERS detection difficult.

Functionalization remains an important step in drawing OCPs close to the SERS substrate. While limiting the diversity of targets that will interact with the plasmonic surface, linkers could be used to attract molecules with similar behavior from complex matrices. For example, lucigenin-modified nanostructures have been used by different researcher groups to detect hydrophobic molecules such as OCPs [24,52] or polycyclic aromatic hydrocarbons (PAHs) [70], but they reported different adsorption profiles for certain molecules. Dithiols have also been reported in the SERS detection of a wide range of hydrophobic organic pollutants such as organochlorine pesticides [23] and aromatic hydrocarbons like anthracene and pyrene [80].

While multiplex analysis of organochlorine pesticides and related molecules is possible in theory, special SERS substrates with large surface areas are needed in order to allow all the molecules to reach the plasmonic surface. Moreover, fingerprint vibrational modes of multiple molecules detected simultaneously (both target and background) would overlap and make their interpretation difficult. Nevertheless, advanced chemometric data analysis could offer a solution in deciphering sample composition from the abundant spectrum information [78].

## 7. Future Outlook/Perspectives

We have already established that SERS could be a much expected and exceptional tool for environmental trace analysis of organochlorine pesticides. A rational design of an active SERS substrate and an appropriate detection strategy must be selected according to the characteristics of the molecular target(s) and the sample matrix. Remarkable efforts have been made to top all the challenges of SERS analysis with obvious progress, but this is hard to accomplish by one particular SERS substrate. Consequently, the inherent features of SERS analysis hyphenated with various other analytical techniques [81] could help solve some of the remaining challenges.

Since sample cleaning or preconcentration is an almost mandatory step preceding SERS analysis of complex samples, coupling SERS with highly selective target analyte capture technologies (molecularly imprinted polymers (MIPs), immunoreactions) might be helpful. MIPs are synthetic polymeric receptors designed to seize a target analyte [82,83]. MIPs coupled with SERS have been used for the ultra-trace detection of various pollutants like bisphenol A [84], TNT (trinitrotoluene) [85], chlorpyrifos [86], or melamine [87] from different matrices. MIPs could also be used as sorbents in solid-phase extraction [88]. SERS-based immunoassay techniques offer, apart from high specificity for the analyte, advantages like high sensitivity and multiplex detection capabilities. Being a biochemical test, it is more often used as a diagnostic tool to detect proteins [89,90], viruses [91,92], or biomarkers [93]. However, immunoreactions have also been designed for DDT detection [94]. A competitive dipstick immunoassay, based on anti-DDT antibodies conjugated with AuNPs was developed for the colorimetric detection of this pollutant from food samples (grapes, cauliflower, milk, and mango juice). When DDT is present in the sample, it is captured by the anti-DDT antibodies and the intensity of the produced color is proportionally reduced. This could be easily coupled with SERS for more reliable results, confirming the nature of the analyte and avoiding unwanted cross-reactivities and inter-batch variabilities of antibodies.

Microfluidic lab-on-a-chip systems are mainly coupled with SERS in order to overcome one of its major drawbacks, namely the reproducibility, by means of mixing confluent streams of analytes and nanostructures in microfluidic channels [95]. Moreover, continuous flow microfluidics could preconcentrate the analyte at the detection area, structure on-site nanoparticle aggregates, and efficiently collect the SERS signal. Hydrophobic molecules like polychlorinated biphenyls could be trace-detected via this technique [96]. Apart from the advantage of using small sample volumes, using integrated microfluidic devices, real-time, rapid, and multiplex analysis could be achieved [97]. One major challenge of in-flow SERS detection remains, improving the interaction between the metallic surface and the analyte.

By combining SERS with electrochemistry, the possibility of using electric fields to attract molecules of different charges is exploited, achieving high analytical selectivity [98] as well as trace detection after electrostatic preconcentration, which was effectively applied for simultaneous detection of minute amounts of polar pollutants [99]. As long as no unwanted or uncontrolled oxidation/reduction processes occur, it is expected that electric fields could be used to position molecules precisely at the SERS active sites, bringing us one step closer toward the much sought single-molecule SERS analysis. Moreover, this spectro-electrochemical hyphenation could be used for in situ monitoring of (electro) chemically-driven processes and redox events with structural changes of surface adsorbates or reaction intermediates [100]. If the SERS technique combines rapid and real-time analysis with label-free target recognition, the spectro-electrochemical sensor could be used for selective/ultrasensitive pesticide detection.

Aspiring for miniaturized systems for on-site trace detection, Mu et al. redesigned the Raman optical system, integrating Raman probes, lenses, and spectrometers together in a cellphone adapted system. Taking advantage of the supra-technologized era, they could simply and rapidly detect 12 pesticide residues including DDT, with a LOD of 10 ppm. This could readily depict the future of on-site SERS analysis regarding environmental pollutants [101].

## 8. Conclusions

OCPs are organic contaminants that persist in the environment for decades after usage, threatening the health of every living organism, along with the equilibrium of ecosystems. Global efforts are directed toward phasing out this harmful class of pesticides, demanding continuous monitoring from complex matrices. In this context, fast, reliable, sensitive, and cost-effective methods of detection and quantification such as SERS are required. SERS exhibits other desirable advantages such as high throughput capabilities and rapid on-site detection of trace amounts of target analytes from complex samples with little to no purification needed. However, SERS analysis of OCPs might be limited by their low affinity toward the substrates or the poor analytical reproducibility often reported for this technique. Nevertheless, in the current review, the most recent ingenious strategies dealing with the mentioned challenges and demonstrating improved SERS detection of OCPs have been presented. Additionally, topics like multiplex analysis or hyphenation of SERS with other instrumental techniques like microfluidics or electrochemistry have also been considered as an additional means of surpassing inherent drawbacks. SERS is rapidly moving from theoretical applications toward real analytical ones, with miniaturized portable systems being developed constantly for specific applications.

## Figures and Tables

**Table 1:** Surface enhanced Raman spectroscopy (SERS) strategies for Organochlorine pesticide (OCP) detection.

Strategy	Analyte	SERS Substrate/Method	LOD	EF	Metal	Laser (nm)	Incubation Time	Ref.
Increasing the substrate’s performance	2,4-D	Au nanorods and Ag nanocubes	-	-	Au, Ag	632.8	1 h	[40]
		vertically ordered arrays of Ag nanorod bundles	61.9 nM	1.4 × 10^8^	Ag + Au	633	hours	[41]
	DDT	void@AuNPs@SiO_2_ microporous capsules	-	-	Au	785	15 min	[42]
		AgNP@composite agarose gels	-	-	Ag	785	2 h	[18]
		AuNP array fabricated by laser annealing of gold film	-	-	Au	785	-	[43]
		AgNPs prepared by self-assembly/ in situ growing method	-	3.5 × 10^6^	Ag	785	<1 min	[44]
		AgNPs sheet	-	-	Ag	632.8	20 min	[45]
	Endosulfan	AuNPs	-	-	Au	785	-	[46]
	HCB	3D Ag F-NPs	-	-	Ag	785	30 min	[47]
	Lindane	chestnut-like Au nanocrystals-built film	34.38 nM	> 10^7^	Au	785	8h	[19]
		concave trisoctahedral and calyptriform Au nanocrystals-built films	0.1 µM	10^7^	Au	785	8h	[48]
		AgNPs sheet	0.3 µM	-	Ag	632.8	20 min	[45]
	PCP	MoO_2_nanodumbbells	0.1 µM	3.75 × 10^6^	MoO_2_	532.8	20 min	[49]
		Fe_3_O_4_xAgNPs@pNIPAM	1 nM	-	Ag	785	2 h	[50]
	>100 pesticides	electrochemically roughened silver oxide SERS sensor	-	-	Ag	785	-	[51]
Increasing the affinity of the analyte	Aldrin	AgNPs@ α, ω-aliphatic diamines	13.7 nM	2 × 10^4^	Ag	514.5	-	[21]
		AuNPs/AgNPs@alkyl dithiols	0.12 µM	-	Au, Ag	785	10 min	[23]
		AgNPs clusters by α, ω-aliphatic diamines	10 nM	-	Ag	785 514.5	-	[22]
		flower like AgNPs@ diquat/lucigenin	-	-	Ag	532	5 min	[52]
	Dieldrin	AuNPs/AgNPs@alkyl dithiols	0.82 µM	-	Au, Ag	785	10 min	[23]
	*op*’-DDT	flower like AgNPs@ diquat/lucigenin	-	-	Ag	532	5 min	[52]
	*pp*’-DDE	flower like AgNPs@ diquat/lucigenin	-	-	Ag	532	5 min	[52]
	Endosulfan (α)	AuNPs/AgNPs@alkyl dithiols	0.41 µM	-	Au, Ag	785	10 min	[23]
	Endosulfan (α, β)	AgNP clusters by α, ω-aliphatic diamines	10nM	-	Ag	785 514.5	-	[22]
	Endosulfan	AgNP@ bis-acridinium lucigenin	49.15 nM	-	Ag	785	-	[24]
	Endosulfan (α, β)	flower like AgNPs@diquat/lucigenin	-	-	Ag	532	5 min	[52]
	HCH	Au nanosheets built hollow sub-microcubes@4-MPBA	1.03 nM	-	Au	785	4h	[53]
	HCH (α, ϒ)	urchin-like Au–Ag nanocrystals@ porous zeolite imidazole framework	15.15 nM	3 × 10^7^	Au + Ag	785	10 h	[25]
	HCH (α, β)	flower like AgNPs@diquat/lucigenin	-	-	Ag	532	5 min	[52]
	HCH (ϒ)	AuNPs/AgNPs@alkyl dithiols	3.53 µM	-	Au, Ag	785	10 min	[23]
	Heptachlor	flower like AgNPs@ diquat/lucigenin	-	-	Ag	532	5 min	[52]
	PCP	AgNPs aggregates@cysteamine SAM	0.20 μM	-	Ag	785	3 h	[54]
		AuNPs@cysteamine	1 nM	5.7 × 10^5^	Au	785	-	[55]
		nanoporous Ag coating@cysteamine	6.4 nM	3.7 × 10^5^	Ag	785	5 h	[56]
		Fe_3_O_4_@carbon@AgNPs core–shell microspheres	1 pM	-	Ag	633	1 h	[57]
	Tetradifon	flower like AgNPs@ diquat/lucigenin	-	-	Ag	532	5 min	[52]
Preconcentra-tion	Chlordane	citrate coated AuNPs/rolling method and prediction model	1 ppm	-	Au	780	-	[58]
	PCP	nanoporous Ag coating modified by cysteamine	6.4 nM	3.7 ×10^5^	Ag	785	5 h	[56]

LOD—limit of detection; EF—enhancement factor; AuNPs—gold nanoparticles; AgNPs—silver nanoparticles; F-NPs—ferro-nanoparticles; SAM—self-assembled monolayer.

## Abbreviations

2,4-D	2,4-dichloro-phenoxyacetic acid
DDE	dichloro-diphenyl-dichloro-ethylene
DDT	dichloro-diphenyl-trichloroethane
DT8	1,8-octanedithiol
EF	enhancement factor
F-NPs	ferro-nanoparticles
GC/ECD	gas chromatography/electron capture detection
GC/MS	gas chromatography/mass spectrometry
HCB	hexachlorobenzene
HCH	hexachlorocyclohexanes
ϒ-HCH	lindane
LOD	limit of detection
LSPR	localized surface plasmon resonance
MIPs	molecularly imprinted polymers
MOFs	metallic organic frameworks
OCPs	organochlorine pesticides
PAHs	polycyclic aromatic hydrocarbons
PCP	pentachlorophenol
pNIPAM	poly(N-isopropylacrylamide)
POPs	persistent organic pollutants
SAM	self-assembled monolayer
SERS	surface enhanced Raman spectroscopy
